# Serum high concentrations of homocysteine and low levels of folic acid and vitamin B_12_ are significantly correlated with the categories of coronary artery diseases

**DOI:** 10.1186/s12872-017-0475-8

**Published:** 2017-01-21

**Authors:** Yan Ma, Duanliang Peng, Chenggui Liu, Chen Huang, Jun Luo

**Affiliations:** 1 East Branch, Sichuan Academy of Medical Sciences & Sichuan Provincial People’s Hospital, No. 585 Hong He North Road, Longquan District Chengdu, 610101 China; 2 Department of Clinical Laboratory, Chengdu Women’s and Children’s Central Hospital, Chongqing Medical University, No. 1617 Ri Yue Avenue, Qingyang District, Chengdu, 610091 China

**Keywords:** Homocysteine, Folic acid, Vitamin B_12_, Coronary artery disease, Atherosclerosis, Endothelial dysfunction

## Abstract

**Background:**

Homocysteine (Hcy) has been considered as an independent risk factor for coronary artery disease (CAD). Folic acid and vitamin B_12_ are two vital regulators in Hcy metabolic process. We evaluated the correlations between serum Hcy, folic acid and vitamin B_12_ with the categories of CAD.

**Methods:**

Serum Hcy, folic acid and vitamin B_12_ from 292 CAD patients, including 73 acute myocardial infarction (AMI), 116 unstable angina pectoris (UAP), 103 stable angina pectoris (SAP), and 100 controls with chest pain patients were measured, and the data were analyzed by SPSS software.

**Results:**

Compared to SAP patients, patients with AMI and UAP had higher Hcy levels with approximately average elevated (4-5) μmol/L, while SAP patients were approximately higher 8 μmol/L than controls. However, the levels of folic acid and vitamin B_12_ had opposite results, which in AMI group was the lowest, while in controls was the highest. CAD categories were positively correlated with Hcy (r = 0.286, *p* < 0.001), and negatively correlated with folic acid (r = -0.297, *p* < 0.001) and vitamin B_12_ (r = -0.208, *p* < 0.001). There were significant trend toward increase in the prevalence of high Hcy, low folic acid and vitamin B_12_ from controls, to SAP, to UAP, and to AMI.

**Conclusions:**

The present study provide the valuable evidence that high concentrations of Hcy and low levels of folic acid and vitamin B_12_ are significantly correlated with CAD categories.

## Background

Coronary artery disease (CAD) is seriously to harm people’s healthy disease in borth developed and developing countries, which was predominantly caused by atherosclerosis with endothelial dysfunction [1, 2]. Despite best efforts, available therapies protect only 30–40% of individuals at risk, and no therapeutic cure is anticipated for those who currently suffer from the disease [3]. The endothelium is a single layer of cells lining all blood vessels. It plays an important role in many physiological functions, including the control of blood cell trafficking, vasomotor tone, vessel permeability, and hemostatic balance. Endothelial cells produce a wide variety of substances in response to various physical and chemical stimuli, including vasodilator substances, and vasoconstrictor substances [4].

Researches have confirmed that endothelial dysfunction, as an impairment of endothelium-dependent relaxation of blood vessels, occur as the initial event in the pathogenesis of atherosclerosis, which considered to be the initiating factor and the key point of cardiovascular disease [5, 6]. Moreover, endothelial dysfunction also play the important role in all stages and categories of CAD from stable angina pectoris (SAP) to unstable angina pectoris (UAP), and to acute myocardial infarction (AMI) [7]. Early warning and immediate risk stratification of patients with different categories of CAD is frequently a challenging task in the current.

A large number of studies have confirmed that serum high homocysteine (Hcy) concentration (morm than 15 μmol/L), which is called hyperhomocysteinemia (HHcy), has been associated with endothelial dysfunction of atherosclerotic CAD owing to oxidative stress [8], endoplasmic reticulum stress [9], involved inflammation [10], increased level of asymmetric dimethylarginine (ADMA) [11] and so on [12–14]. Elevated ADMA can results in decreasing endothelium-derived nitric oxide concentration and bioavailability [15]. Nitric oxide as a most important mediator of endothelium-dependent relaxation, is a potent vasodilator, which plays a key role in normal vascular physiology in preserving the vessel wall in a quiescent state by inhibition of inflammation, thrombosis, and cellular proliferation [16]. Decreased nitric oxide bioavailability would result in the abnormal thrombosis, vasorelaxation, and atherosclerosis, thereby promoting the occurrence and development of CAD [17].

Folic acid and vitamin B_12_ play an important role in regulating the metabolic process of Hcy [18]. Current studies have shown that supplement folic acid, vitamin B_12_ in patients with HHcy could reduce Hcy levels [19]. Folic acid supplementation not only may be useful in reducing Hcy level in high risk patients with HHcy [20], but also can significantly improve endothelial dysfunction in patients with CAD [21]. On the other hand, folic acid deficiency or/and vitamin B_12_ deficiency would result in HHcy [22–24]. Vitamin B_12_ deficiency and HHcy are related to cardiovascular risk factors in patients with CAD [25]. However, the correlations of CAD categories with Hcy, folic acid, vitamin B_12_ have not been reported. Therefore, we evaluate the correlations between CAD categories and each of the metabolic parameters, anthropometric variables, life style habits and traditional cardiovascular risk factors in CAD patients and controls with chest pain patients.

## Methods

This study included 292 CAD patients (203 male and 89 female) aged 36–85 (62.54 ± 14.52) years, and 100 controls with chest pain symptom (69 male and 31 female) aged 38–87 (60.93 ± 15.65) years, from the Sichuan Academy of Medical Sciences & Sichuan Provincial People’s Hospital. There were no statistically significant difference in age (means ± SD, t = 0.94, *p* = 0.348) and gender (male to female ratio, *χ* ^2^ = 0.01, *p* = 0.922) between CAD group and controlled group.

All enrolled CAD patients had been confirmed by coronary angiography and were diagnosed to be103 SAP, 116 UAP, 73 AMI according to 2007 ACC/AHA guidelines. 100 controls with chest pain patients in the same period were confirmed by coronary angiography too. Patients with the following diseases were excluded from this study: cancer, liver diseases, renal insufficiency, blood diseases, hyperthyroidism, thyroid dysfunction, systemic lupus erythematosus, malnutrition, pregnant woman, and supplemented folic acid and vitamin B_12_.

Participating subjects were explained their participation rights and written informed consent was obtained, and were asked about alcohol intake situation (yes or no, it was defined as yes at least once a week and drinking over 45° of alcohol more than 200 mL) and smoking habits (yes or no, non-smokers including never smoking and stop smoking more than 1 year).

The data was consecutively collected from October 2013 to September 2014. Fasting blood was sampled in the morning within 24 h that the patients had been admitted to hospital. The blood must be collected before the heparinization of coronary angiography. Moreover, AMI patient’s blood was collected before percutaneous coronary intervention and thrombolytic treatment. After blood was separated, a fresh serum were used with Hitachi 7600 Automatic Biochemistry Analyzer (Hitachi High-Tech Instruments Co., Ltd., Japan) for the determinations of Hcy, total cholesterol (TC), triglyceride (TG), high density lipoprotein cholesterol (HDL-C), low density lipoprotein cholesterol (LDL-C), glucose (GLU) and uric acid (UA). Another fresh serum were used for the determinations of folic acid and vitamin B_12_ by ACCESS 2 Immunoassay System (Beckman Coulter, Inc., USA).

High Hcy, folic acid and vitamin B_12_ were defined as Hcy, folic acid and vitamin B_12_ greater than 15 μmol/L, 26.0 nmol/L and 675 pmol/L, respectively, while low Hcy, folic acid and vitamin B_12_ were defined as Hcy, folic acid and vitamin B_12_ less than 5 μmol/L, 6.8 nmol/L and 133 pmol/L, respectively, according to their references intervals were (5–15) μmol/L for Hcy, (6.8–26.0) nmol/L for folic acid and (133–675) pmol/L for vitamin B_12_, respectively. Systolic blood pressure (SBP) and diastolic blood pressure (DBP), body weight and height were measured with standard techniques. Body mass index (BMI) was calculated as body weight (kg) divided by the square of height (m).

Hypercholesterolemia and hypertriglyceridemia were defined as TC ≥ 6.22 mmol/L and TG ≥ 2.26 mmol/L, respectively, according to 2007 China Adult Dyslipidemia Prevention Guide. Diabetes mellitus was diagnosed when patients’ GLU ≥ 7.0 mmol/L. Hypertension was diagnosed when patients’ SBP ≥ 140 mmHg or DBP ≥ 90 mmHg. Overweight and obesity were defined as BMI (24.0–27.9) kg/m^2^, and ≥ 28 kg/m^2^, respectively, according to 2006 Guidelines for Prevention and Control of Overweight and Obesity in Chinese Adults.

### Statistical analysis

The data were analyzed by using the statistical package for social science SPSS software version 16.0 (SPSS, Inc., Chicago, IL, USA). Continuous variables were expressed as mean ± SD because the data presented in this study showed a normal distribution. Means ± SD of two samples were compared by the Independent-Sample *t*-Test, and means ± SD of more than two samples were compared with the One-Way ANOVA. Categorical variables were expressed as percentage and compared by *χ* ^2^-test. The correlation coefficients of CAD categories with each of the metabolic parameters, anthropometric variables and life style habits were calculated by Spearman’s analysis because CAD is a grade variable, while the correlation study between Hcy and folic acid as well as vitamin B_12_ were performed on the measured data by using Pearson’s correlation a coefficient because Hcy, folic acid and vitamin B_12_ are continuous variables with normal distribution. A *p*-value < 0.05 was considered as significant.

## Results

### Comparison of principal characteristics between high Hcy, normal Hcy and low Hcy levels in CAD patients

Compared to normal and low Hcy groups, High Hcy group were characterized by smoking, Diabetes mellitus, hypercholesterolemia, hypertriglyceridemia, low folic acid, low vitamin B_12_, low HDL-C and high LDL-C (*p* < 0.05). There were no significant differences in the ratio of elder age, male, female, alcohol drinking, hypertension, overweight and obesity among three groups. The comparison of principal characteristics between high Hcy, normal Hcy and low Hcy levels in 292 CAD patients are reported in Table 1.

**Table 1:** Comparison of principal characteristics between high Hcy, normal Hcy and low Hcy levels in 292 CAD patients

	High Hcy group (*n* = 231)	Normal Hcy group (*n* = 58)	Low Hcy group (*n* = 3)	*χ* ^2^	*p* value
Elder age ≥ 61 (%)	61.47	51.72	33.33	2.67	0.264
Male (%)	71.00	63.79	66.67	1.15	0.564
Female (%)	29.00	36.21	33.33	1.15	0.564
Smoking (%)	29.87	13.79	33.33	6.19	0.045
Alcohol drinking (%)	39.39	34.48	66.67	1.47	0.479
Hypertension (%)	63.20	56.90	33.33	1.81	0.405
Diabetes mellitus (%)	30.30	13.79	33.33	6.46	0.039
Hypercholesterolemia (%)	46.32	25.86	0	10.15	0.006
Hypertriglyceridemia (%)	30.74	13.79	66.67	8.93	0.012
Overweight and Obesity (%)	40.26	44.83	33.33	0.48	0.789
Low folic acid (%)	51.08	6.90	0	39.39	<0.001
Low vitamin B_12_ (%)	41.99	8.62	0	24.34	<0.001
Low HDL-C (%)	38.96	20.69	0	8.44	0.015
High LDL-C (%)	39.83	22.41	0	7.81	0.020

More than half of the CAD patients (51.08%, 118/231) with high Hcy had low folic acid levels, 7 times higher than that (6.56%, 4/61) in CAD patients with normal-low Hcy concentrations (*p* < 0.001), and 41.99% (97/231) CAD patients with high Hcy had low vitamin B_12_ levels, 5 times higher than that (8.20%, 5/61) in CAD patients with normal-low Hcy concentrations (*p* < 0.001).

### Comparison of Hcy, folic acid and vitamin B_12_ between CAD and controls

AMI patients had the highest serum concentrations of Hcy, and UAP patients were a little lower than AMI but were the second highest. and SAP patients had the third higher level of Hcy, which were significantly higher than controls (*p* < 0.001). Compared to SAP patients, patients with AMI and UAP had higher Hcy levels with approximately average elevated (4-5) μmol/L, while SAP patients were approximately higher 8 μmol/L than controls. However, the levels of folic acid and vitamin B_12_ had opposite results, which in AMI group had the lowest, while in controlled group had the highest. The comparison of Hcy, folic acid and vitamin B_12_ between AMI, UAP, SAP groups and controls are shown in Table 2.

**Table 2:** Comparison of Hcy, folic acid and vitamin B_12_ between AMI, UAP, SAP patients and controlled group with chest pain patients

Groups	Number	Hcy (μmol/L)	Folic acid (nmol/L)	Vitamin B_12_ (pmol/L)
Controls	100	10.81 ± 4.62	12.86 ± 5.85	222.34 ± 62.58
Stable angina pectoris	103	18.63 ± 6.73^a^	10.33 ± 4.95^b^	167.52 ± 56.25^b^
Unstable angina pectoris	116	22.62 ± 6.37^ac^	9.21 ± 4.38^bd^	148.65 ± 62.51^bd^
Acute myocardial infarction	73	23.44 ± 5.78^ac^	7.08 ± 3.43^bde^	144.57 ± 52.24^bd^
F		90.51	22.05	35.63
*p* value		<0.001	<0.001	<0.001

^a^Significantly increased compared to controls, ^b^Significantly decreased compared to controls, ^c^Significantly increased compared to SAP group, ^d^Significantly decreased compared to SAP group, ^e^Significantly decreased compared to UAP group

### Correlation coefficients of CAD categories with each of the variables and the correlations between Hcy with folic acid and vitamin B_12_

The correlation coefficients of CAD categories with each of the metabolic parameters, anthropometric variables and life style habits by Spearman’s analysis in 292 CAD patients are shown in Table 3. CAD categories were positively correlated with Hcy, TC, TG, LDL-C, age, SBP, DBP, BMI, gender and smoking, and negatively correlated with folic acid, vitamin B_12_ and HDL-C levels. On the contrary, CAD categories were not significantly correlated with GLU, UA and alcohol drinking. Among them, Hcy and folic acid showed the highest positively and negatively correlated with CAD categories, respectively.

**Table 3:** Spearma’s correlation coefficients of CAD categories with each of the metabolic parameters, anthropometric variables and life style habits in 292 CAD patients

Variable	CAD categories	*p* value
Homocysteine	0.286	<0.001
Folic acid	-0.297	<0.001
Vitamin B_12_	-0.208	<0.001
TC	0.242	<0.001
TG	0.141	0.016
HDL-C	-0.153	0.009
LDL-C	0.187	0.001
GLU	0.088	0.132
UA	0.078	0.182
Age	0.151	0.010
SBP	0.135	0.021
DBP	0.125	0.032
BMI	0.148	0.011
Gender	0.128	0.028
Smoking	0.278	<0.001
Alcohol drinking	0.072	0.218

*Hcy* homocysteine, *TC* total cholesterol, *TG* triglyceride, *HDL-C* high-density lipoprotein cholesterol, *LDL-C* low-density lipoprotein cholesterol, *GLU* glucose, *UA* uric acid, *SBP* systolic blood pressure, *DBP* diastolic blood pressure, *BMI* body mass index

Pearson’s correlation analysis showed that there were strongly moderate negative correlations between Hcy and folic acid (r = -0.666, *p* < 0.001) and vitamin B_12_ (r = -0.564, *p* < 0.001).

### Prevalence of high Hcy, and low folic acid and vitamin B_12_ in CAD patients and controls with chest pain patient

**Fig. 1:**
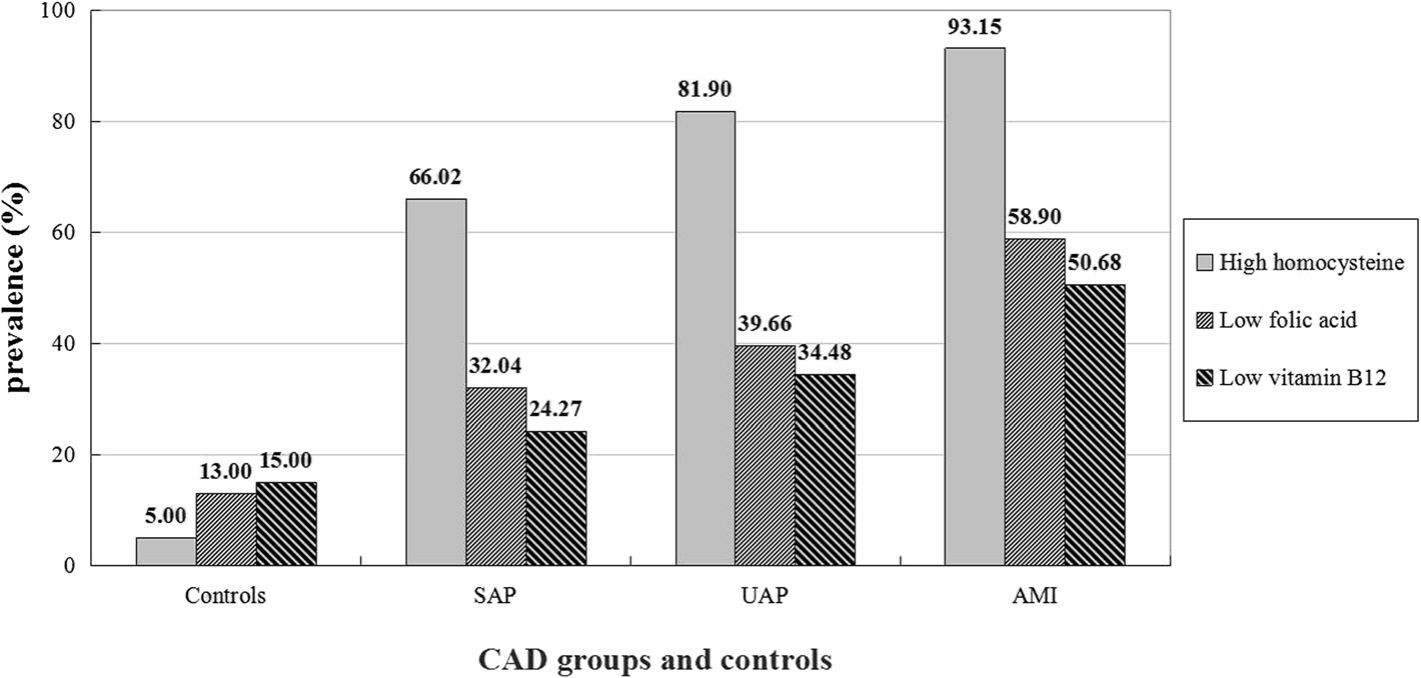
Prevalence of high Hcy, low folic acid and vitamin B_12_ in 292 CAD patients and 100 controls with chest pain patient aged 36–87 years

Approximately four-fifths of CAD patients (79.11%, 231/292) had a prevalence of high Hcy. However, the levels of folic acid and vitamin B_12_ in CAD patients were reduced, the prevalence were 41.78% (122/292) for folic acid, and 34.93% (102/292) for vitamin B_12_, respectively. The prevalence of high Hcy, and low folic acid and vitamin B_12_ in 292 CAD patients and 100 controls with chest pain patient are shown in Table 4 and Fig. 1, respectively. There was a significant trend toward an increase in the prevalence of high Hcy from controls, to SAP, to UAP, and to AMI. The prevalence of high Hcy progressively increased from 5.00% in controls, to 66.02% in SAP group, to 81.90% in UAP group, and to 93.15% in AMI group (*p* < 0.001). Low folic acid and vitamin B_12_ also had significant trend toward rise in the prevalence from controls, to SAP, to UAP, and to AMI. The prevalence of low folic acid progressively increased from 13.00% in controls, to 32.04% in SAP group, to 39.66% in UAP group, and to 58.90% in AMI group, respectively (*p* < 0.001). Similarly, the prevalence of low vitamin B_12_ progressively increased from 15.00% in controls, to 24.27% in SAP group, to 34.48% in UAP group, and to 50.68% in AMI group, respectively (*p* < 0.001).

**Table 4:** The prevalence of high Hcy, low folic acid and vitamin B_12_ in 292 CAD patients and 100 controls with chest pain patient (%)

	Number	High Hcy (*n* = 236)	Low folic acid (*n* = 135)	Low vitamin B_12_ (*n* = 117)
Controls	100	5.00	13.00	15.00
Stable angina pectoris	103	66.02	32.04	24.27
Unstable angina pectoris	116	81.90	39.66	34.48
Acute myocardial infarction	73	93.15	58.90	50.68
*χ* ^2^		184.51	41.37	28.39
*p* value		<0.001	<0.001	<0.001

## Discussion

CAD is a multifactorial disease, and these factors are called risk factors include a large number of traditional cardiovascular disease risk factors such as smoking, elder age, male, hypertension, lipid metabolism disorders (hyperlipidemia), glucose metabolism disorders (diabetes mellitus and insulin resistance), overweight and obesity, and some newly risk factors such as HHcy and inflammatory markers. Our previous study found that elevated serum myeloperoxidase activities are significantly associated with the prevalence of acute coronary syndrome (AMI and UAP) and high LDL-C levels in CAD patients, The interaction between multiple metabolic parameters, inflammatory markers and traditional cardiovascular risk factors promoted the occurrence and development of CAD [26]. This study revealed that high Hcy group in CAD patients were characterized by smoking, diabetes mellitus, hypercholesterolemia, hypertriglyceridemia, low HDL-C and high LDL-C. These findings suggest that Hcy and traditional cardiovascular risk factors may be synergistically prompt the occurrence and development of CAD.

Vascular endothelium has important regulatory functions in the cardiovascular system and a pivotal role in regulating blood flow, mediating vasodilatation, coagulation reactions, platelet activation, leukocyte adhesion, and vascular muscle function [27]. During the last two decades, extensive experimental evidence, both in vitro and in vivo, indicates that Hcy is an independent risk factor for cardiovascular disease and elevated serum Hcy level is associated with CAD events [28–30]. Homocysteine Studies Collaboration research revealed that elevated approximately 3 μmol/L Hcy will increase about 10% risk of cardiovascular events [31]. Humphrey et al. [32] analyzed has also demonstrated that increased 5 μmol/L Hcy concentration will increase approximately 20% risk of CAD events.

In present study, AMI patients had the highest serum concentrations of Hcy, and UAP patients were a little lower than AMI but were the second highest, and SAP patients had the third higher level of Hcy, which were significantly higher than controls. Compared to SAP patients, patients with AMI and UAP had higher Hcy levels with approximately average elevated (4-5) μmol/L, while SAP patients were approximately higher 8 μmol/L than controls. CAD categories were positively correlated with Hcy, TC, TG, LDL-C, age, SBP, DBP, BMI, gender and smoking. Among them, Hcy showed the highest positively correlated with CAD categories. The prevalence of high Hcy progressively increased from controls, to SAP, to UAP, and to AMI. The present provide the valuable evidence that high concentrations of Hcy are significantly correlated with CAD categories. The more serious patients with CAD suffer, the more higher concentration their Hcy have.

Folic acid and vitamin B_12_ as two vital regulators play an important role in regulating the metabolic process of Hcy [33, 34]. In biological cells, Hcy is derived from methionine after its utilization as a methyl group donor in biological methylation reactions. However, approximately 50% Hcy is produced to remethylate back to methionine by the transmethylation of methionine, while 50% Hcy metabolize via transsulfuration to cystathionine [35]. In this cycle, methionine is activated by condensation with adenosine triphosphate (ATP) to give the methyl donor, S-adenosylmethionine (SAM). SAM is transformed into S-adenosylhomocysteine (SAH) by donating its methyl group to the substrates of methylation reactions. Subsequently, SAH gives rise to Hcy in a reversible reaction that favors SAH over Hcy production [36]. Methyl-tetra-hydrofolic acid (MTHF) which derivate of folic acid provide methyl to remethylated of Hcy. Vitamin B_12_ is agon of methionine synthetase that catalyzed this reaction and participate transfusion of methyl [13]. Folic acid deficiency will prevent remethylation of Hcy because of raw material deficiency. Moreover, Folic acid deficiency will also influence the production of MTHF through to affect activity of methylene tetrahydrofolate reductase (MTHFR) [37, 38].

We found that besides HDL-C, CAD categories were significantly negative correlated with folic acid, vitamin B_12_. the levels of folic acid and vitamin B_12_ in AMI and in UAP patients were obviously lower compared to those in SAP and controls. The prevalence of low folic acid and vitamin B_12_ progressively increased from controls, to SAP, to UAP, and to AMI. Moreover, more than or close to half of the CAD patients with high Hcy had low folic acid or vitamin B_12_ levels, 7 times or 5 times higher than that in CAD patients with normal-low Hcy concentrations, respectively. Hcy was strongly moderate negative correlation with folic acid and vitamin B_12_. Our results confirmed that serum folic acid and vitamin B_12_ influence Hcy metabolism as cosubstrate and cofactor, respectively. Low serum levels of folic acid and vitamin B_12_ are also significantly correlated with CAD categories.

### Study limitation

Since present study was just an investigation that the correlations between CAD categories and serum Hcy, folic acid, vitamin B_12_ and traditional cardiovascular risk factors. The larger sample number of multicenter study and longer prospective investigation are necessary to further observe serum Hcy changes and incidence of adverse cardiovascular events by supplementation of folic acid and vitamin B_12_ in CAD patients.

## Conclusions

The present study confirmed that Hcy and traditional cardiovascular risk factors may be synergistically prompt the formation and development of atherosclerosis in CAD patients. High concentrations of Hcy and low levels of folic acid and vitamin B_12_ are significantly correlated with CAD categories.

## Abbreviations

ADMA: Asymmetric dimethylarginine
AMI: Acute myocardial infarction
ATP: Adenosine triphosphate
BMI: Body mass index
CAD: Coronary artery disease
DBP: Diastolic blood pressure
GLU: Glucose
Hcy: Homocysteine
HDL-C: High density lipoprotein cholesterol
HHcy: Hyperhomocysteinemia
LDL-C: Low density lipoprotein cholesterol
MTHF: Methyl-tetra-hydrofolic acid
MTHFR: Methylene tetrahydrofolate reductase
NO: Nitric oxide
SAH: S-adenosylhomocysteine
SAM: S-adenosylmethionine
SAP: Stable angina pectoris
SBP: Systolic blood pressure
TC: Total cholesterol
TG: Triglyceride
THF: Ttrahydro-folic acid
UAP: Unstable angina pectoris
